# From first to last author: evaluation of women’s career progression in mental health publishing in one institution

**DOI:** 10.1192/bjo.2025.10931

**Published:** 2025-12-15

**Authors:** Daniel Stahl, Imogen Kilcoyne, Phil Staite, Til Wykes

**Affiliations:** Institute of Psychiatry, Psychology and Neuroscience, King’s College London, London, UK

**Keywords:** Gender, career progression, academia, publications, mental health

## Abstract

**Background:**

There is ample evidence that women do not progress in mental health publishing as quickly as men. The movement from first to last (senior) author is one indicator of progression.

**Aims:**

To understand whether there are changes in women’s authorship position following our academic institution’s introduction of support mechanisms to reduce the gender gap in career development.

**Method:**

Data from publicly held databases in three cohorts (2016, 2018 and 2020) were assessed for gender and authorship position at the Institute of Psychiatry, Psychology and Neuroscience. Regression analyses included authorship gender and change over time in authorship roles, by school and topic.

**Results:**

We found substantial, statistically significant differences in gender between author roles (χ^2^(2) = 29.18, *P* < 0.0001), with women being mainly first authors (marginal mean 62.2:40.1%, respectively, odds ratio 2.463, 95% CI 1.807 to 3.357). The three schools differed (χ^2^(2) = 14.06, *P* < 0.001) and, although men were predominant as last authors in all topics in both 2016 and 2020, women did show a modest increase. The trend for an interaction between gender and first-author publications on the likelihood of last-author publications in 2018 (incidence rate ratio 1.839, 95% CI 0.914 to 3.698) had disappeared by 2020.

**Conclusions:**

Although women were represented as first and corresponding authors, there was still a gender gap for last-author positions. Over time, women have increased their representation in many of the topic areas. The disappearance of any gender-moderating effect suggests that institutional policies may have had an effect, in addition to sector-wide changes.

Research is dependent on a diverse research team but there remains a gender gap in careers, with women less well represented in publication output. This paper investigates one indicator for potential career progression: the representation as first, corresponding or last author of publications. We used publications from the largest UK-based mental health research institution and found that some progress has been made, but there remains a gap, especially in the representation as last authors and the move from first to last (usually senior) author. This is despite the institution’s support for reducing gender barriers.

For health sciences, women’s authorship position reflects their career progression, especially the transition from first to last (usually senior) author. With the employment of women in mental health sciences increasing, this should have had an impact on the change to senior author position. However, this often depends on the nature of the organisation, availability of role models, influence of support for career development and its management of diversity.^1–3^ Wykes and colleagues^4^ demonstrated that movement from first to last author was sluggish, with the rate of change for psychology and psychiatry women authors for every 10 years being 8.6% for first and 6.9% for last author, although this rate was higher than in papers in medicine (2.4 and 2.7%, respectively). The 2024 volume of the *British Journal of Psychiatry* showed proportions of first and last authors similar to those found in the review.^4^ There have been many initiatives aimed at tackling this issue but new research has shown that gender inequality persists, with men still dominating scientific production.^5^ Reversal of advances has also been identified, such as a reduction in women authors during COVID-19,^6^ so it is vital to keep reviewing the situation and changing or strengthening policies when indicated.

## Changing the gender disparity

In the UK, research funders have strongly encouraged universities to utilise the Athena Swan award framework to identify and transform gender equality within higher education.^7^ The Institute of Psychiatry, Psychology and Neuroscience (IoPPN) at King’s College London (KCL) adopted diversity and inclusion as a core value and, over the past decade, there have been concerted efforts to increase gender equality. The IoPPN received its first Athena Swan Silver Award in 2014, and again in 2019, based on sustained progress towards gender equality that was supported by training and attention to gender diversity at all academic levels – for instance, the ‘Inspiring Women’ showcase of 31 women professors.

Although the faculty has reached the milestone of 40% of the professoriate being women, this paper aims to identify whether the policies and training advocated were associated effects on the proportions of women authors. We were particularly interested in whether there has been an increase in women in first-, last- or corresponding-author positions between 2016 and 2020, when Athena Swan policies were in place. The choice to limit the search to these years was to stop prior to the COVID-19 pandemic, when there were changes in both publication policies and potential reversals in authorship.^6^ Our aim is to identify whether the policies adopted were associated with a reduced effect of gender on authorship, and to assess the need to implement new systems or supports. We were particularly interested in how gender affects authorship roles and very early career publication, as well as influencing the progression to senior authorship in academic publications at the IoPPN, and whether the topic of research has any effect on this transition.

## Method

### Design

The IoPPN is the largest UK centre for mental health research, and consists of three schools providing multidisciplinary research aimed at understanding, preventing and treating mental illness and other conditions that affect the brain. IoPPN research is highly cited (second globally), reflecting its significant research impact, and publishes widely, so it represents an excellent mental health location for testing of issues of gender equality. All research is multidisciplinary and crosses school boundaries. We analysed publicly accessible databases, and for publications we used the KCL document repository. We extracted papers in three separate cohorts – 2016, 2018 and 2020 – and identified author positions (first, corresponding, last), schools and topics: (a) for unique IoPPN staff and ‘students’ and (b) for publication counts and first-, last- and corresponding-author positions. The initial date was chosen to ensure a high-quality data-set that had been checked for the University Research Excellence Framework, and was implemented following the introduction of a school structure. The data were analysed to understand the relative positions of those who published and, importantly, changes over time.

### Data description and quality

We used the Topics tool in SciVal that categorises publications into research topics based on citation patterns and keyword analysis. Where SciVal could not assign a topic (*n* = 36), the All Science Journal Classification (ASJC) code was manually determined by D.S. and T.W. ASJC codes were then mapped into five topic categories using a predefined hierarchy (see Supplementary Table 1 available at https://doi.org/10.1192/bjo.2025.10931). We examined the different dates held in the repository – accepted/in-press, e-Pub ahead of publication or published – against publication dates held in Scopus, and found that the best match was the ‘published’ date. Where papers listed joint first or last authors, each author was treated as an independent data point in the analyses, and we did not account for their co-authorship on the same paper. A small number of publications (approximately 1%) had a single author, who was counted as both first and last author. These papers do not affect the transition analyses, because such authors would have been excluded if they already had a last-author publication at baseline.

Gender was established by the author’s presentation and use of pronouns on KCL and/or personal websites, as well as via publicly available social media data. This process is less open to challenge than when using algorithms, especially for names originating from non-Western countries.

We ensured that authors had a contractual relationship with IoPPN of at least 12 months prior to the final publication date. For some analyses we included PhD student authors identified as students (Supplementary Table 2), because we wanted to understand whether the process of gender disparities emerged very early. There are specific IoPPN supports for career development and, although these are not gendered, we wanted to understand whether they had had any effects over time. Where we included students as authors in the analyses, we also carried out a sensitivity analysis separating the groups and have included these in the supplementary data.

### Data analyses

Our aims were to: (a) describe women’s authorship roles across publication years and academic positions (staff versus student), while considering the influence of school affiliation; (b) understand whether publishing as first author was associated with progression to senior (last) author, and explore whether this varies by gender and school affiliation; and (c) examine whether gender-moderating effects on the move to senior author are affected by research topic.

Summary statistics describe the gender distribution in authorship roles (first, corresponding and last) across the years 2016, 2018 and 2020, as well as differences between IoPPN schools. To account for skewed distributions, boxplots and bar charts were used to visualise trends over time.

Our analyses were conducted in three parts. In the first of these, a logistic regression with cluster robust standard errors was conducted to examine differences in women’s authorship across the years 2016, 2018 and 2020, author roles (first, corresponding and last) and academic position (staff versus student). The dependent variable was a binary gender indicator (1, woman; 0, man), with time, role and authorship position as categorical covariates. In the next step, we included school as an additional categorical covariate. Due to a limited sample size, only interactions between year and role were assessed and, if negligible (*P* > 0.2), these were excluded from the final model.

In the second part, two negative binomial regression models with robust standard errors were conducted to determine whether first-author publications influenced progression to last authorship. Given the skewed distribution of publication counts, a negative binomial regression was selected over Poisson regression to account for overdispersion in the data.^8^ In the 2016 cohort, the number of last-author publications in 2018 was used as the outcome variable, with the mean-centred number of first-author publications in 2016, gender and their interaction as independent variables. We included only individuals who were not the last author at baseline and remained on contract for at least 1 year before the follow-up period, assuming that publications were typically submitted 1 year before they were published. Similarly, in the 2018 cohort, the number of last-author publications in 2020 was the outcome variable, with first-author publications in 2018, gender and their interaction as predictors. The interaction term between gender and first-author publications was included to test whether gender moderated the relationship between early career authorship and progression to last authorship. School was then added as an independent variable to assess differences between schools. Each cohort was analysed separately, with a potential bias due to the lack of data for the intervening years (2017 and 2019), during which individuals may have published additional papers.

In the third part, descriptive analyses of research topics were performed to explore whether gender differences in research topics influence progression to senior authorship roles. Due to the limited sample size for each topic, these analyses were primarily exploratory and did not allow for robust pattern detection.

For all regression analyses, clustered robust standard errors were applied to address dependency arising from repeated observations of the same individuals.^8^ When sample sizes became very small, no further analyses were carried out. All analyses were conducted using Stata version 18 for Windows, with statistical significance set at *P* < 0.05 and trends at *P* < 0.1.

## Results

### Data-set

The IoPPN published 2459 peer-reviewed research publications during the years 2016, 2018 and 2020, with 803, 797 and 859, respectively, not including editorials or chapters. Of these publications, 183 had joint first authorship and 163 joint last authorship. In regard to gender, we found a small number of authors who did not identify as either a man or a woman (*n* = 3), but we were able to identify the gender of all the other authors, so excluded only these three. Table 1 shows that the three schools were relatively similar in regard to first, last and corresponding authors. Of the 916 IoPPN members designated as first, last and/or corresponding authors, there were 575 different staff as first authors, 523 as corresponding and 365 as last authors. Of this number, 58.4% were women (with most being staff members (81.8%) and the remainder students), who were listed as first authors on 160 papers, as corresponding authors on 81 papers and, as might be expected, with only 7 as last authors. Percentage and other count data for all analyses are shown in Supplementary Tables 3–5).

**Table 1:** Authorship position distribution by school, combined across different year cohorts

Authorship role	Mental health and psychological sciences, *n* (%)	Neuroscience, *n* (%)	Academic psychiatry, *n* (%)
Corresponding	241 (36.9)	141 (21.6)	272 (41.6)
First	291 (36.8)	186 (23.5)	313 (39.6)
Last	141 (35.1)	101 (25.2)	159 (39.7)

### Aim 1: gender differences across role, time and school

**Fig. 1:**
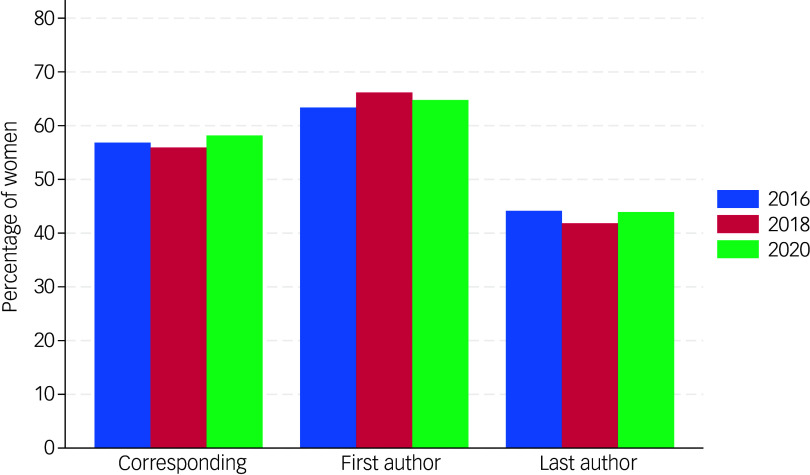
Percentages of women as corresponding, first and last authors, 2016, 2018 and 2020.

Figure 1 shows that the percentage of women authors in each year is highest for the first- and lowest for the last-author position.

A logistic regression, with gender as the dependent variable and time and authorship role as categorical covariates, shows non-significant differences across the years (*χ* ^2^(2) = 1.04, *P* = 0.60). However, differences between roles are substantial and statistically significant (*χ* ^2^(2) = 29.18, *P* < 0.0001). Specifically, the proportion of women first authors (marginal mean, 62.2%) is significantly higher compared with both women last authors (marginal mean 40.1%, odds ratio 2.463 (95% CI 1.807 to 3.357, *z* = 5.70, *P* < 0.0001) and women corresponding authors (marginal mean 54.7%, odds ratio 1.365 (95% CI 1.135 to 1.642, *z* = 3.30, *P* = 0.001). In addition, the proportion of women as corresponding authors is significantly higher than that as last authors (odds ratio 1.804, 95% CI 1.4446 to 2.252, *z* = 5.22, *P* < 0.001). When rank (staff versus student) was added as an additional variable, there was a trend indicating a higher proportion of women among students (61.7%, odds ratio 1.590 (95% CI 0.964 to 2.622, *z* = 1.82, *P* = 0.69) compared with staff (50.3%). The inclusion of rank did not substantially alter the effects of role or time.

A logistic regression, with gender as the dependent variable and role, school and year as categorical covariates, reveals the same pattern, with no statistically significant differences (*χ* ^2^(2) = 1.94, *P* = 0.38), and the pattern was also the same (*χ* ^2^(2) = 34.5, *P* < 0.001). There were significant differences among the three schools (*χ* ^2^(2) = 14.06, *P* < 0.001): mental health and psychological sciences had higher average proportions of women as corresponding, first and last authors in comparison with academic psychiatry and neuroscience (both *P* < 0.01), with no differences between the two disciplines (*P* = 0.63). The marginal means of all three author roles are mental health and psychological sciences 64.2%, academic psychiatry 45.2% and neuroscience 41.6%.

An exploratory assessment of position (staff versus student) did not alter the conclusions of the model, and there were no differences between staff and students (*P* = 0.12).

In summary, women who published papers are overrepresented as first authors and underrepresented as last authors, with corresponding authors falling somewhere in between. This pattern is consistent across schools and remains stable over time, but there are some differences. Students were primarily listed as first authors and, as expected, rarely as last authors, with a higher proportion of women among students than staff.

### Trends in publication counts, by gender and role

On average, the mean number of staff member publications increased over time. For corresponding authors, mean numbers of publications across the year cohorts were 0.658 (range 0–33, *n* = 730), 0.732 (0–25, *n* = 667) and 0.945 (0–14, *n* = 567); for first authorships the numbers were, respectively, 0.493 (0–25), 0.592 (0–23) and 0.732 (0–6); and, for last authorships, 0.922 (0–54), 0.955 (0–32) and 1.25 (0–18), respectively. Students had fewer first-author papers and almost no last-authorship publications (see Supplementary Fig. 1). In the following analyses, students were excluded due to the small sample sizes. Further, similar figures are shown in Supplementary Figs 2–4, grouped by IoPPN schools.

### Aim 2: does gender influence the movement from first to last author?

#### Phase 1: 2016–2018

**Fig. 2:**
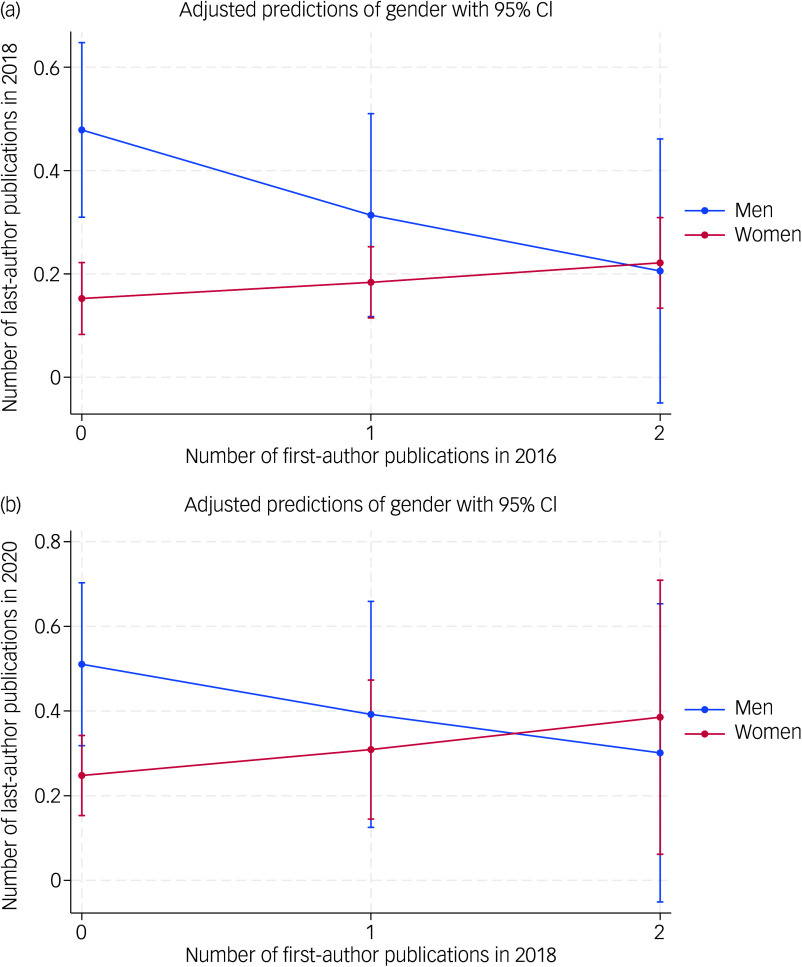
(a) Marginal means (with 95% CI) of predicted last-author publications in 2018, separately for men and women, for 0, 1 and 2 first-author publications in 2016. (b) Predicted publications in 2020 for 0, 1 and 2 first-author publications in 2018.

In 2016 and 2018, 189 men and 283 women had an IoPPN contractual link and had not yet published a last-author paper in 2016. We performed a negative binomial regression, with the number of last-author publications in 2018 as dependent and gender and the number of first-author publications in 2016 and their interaction as independent variables. The analyses revealed a trend for the interaction between gender and number of first-author publications, suggesting a moderating effect of gender (incidence rate ratio (IRR) 1.839 (95% CI 0.914 to 3.698), *z* = 1.71, *P* = 0.087). Figure 2 shows that, with an increasing number of first-author publications in 2016, the gender gap decreases and becomes non-significant (*P* > 0.22 for one and *P* = 0.91 for two publications). Men without any first-author publications in 2016 were more likely than women to become last authors in 2018 (estimated mean difference −0.326, 95% CI −0.509 to −0.143; *χ* ^2^(1) = 12.2, *P* = 0.0005). Figure 2 shows that this gender gap decreases with one or two first-author publications and becomes non-significant (*P* > 0.22 for one and *P* = 0.91 for two publications).

#### Phase 2: 2018–2020

In 2018, 123 men and 193 women did not have a last-author paper in 2016 or 2018 and were still employed at IoPPN in 2020. As in the 2016–2018 analysis, for those who did not publish a first-author publication in 2018, women were less likely to publish as the last author in 2020 (IRR = 0.485 (95% CI 0.284 to 0.683), *z* = −2.64, *P* < 0.008). Figure 2(b) illustrates a narrowing gender gap, with increasing numbers of first-author publications in 2018; the difference is smaller than in the 2016–2018 analysis and is no longer statistically significant (IRR = 1.624, 95% CI 0.839 to 3.144, *z* = 1.44, *P* = 0.15).

Adding ‘school’ as an additional independent variable did not reveal significant differences among schools (2016–2018: *χ* ^2^(2) = 0.66, *P* = 0.72; 2018–2020: *χ* ^2^(2) = 0.42, *P* = 0.82), and the conclusions regarding gender disparities remained largely unchanged for both phases.

### Aim 3: does the research topic affect how gender is represented in authorship?

We classified publications into six topics: (a) medicine, pharmacology and health (MPH), (b) neurology and neuroscience, (c) psychiatry and mental health (PMH), (d) psychology, (e) science and (f) other. Averaged across years, almost half of the first, last and corresponding authors’ publications were, as expected in PMH, followed by neuroscience and MPH (about 16% each), and fewer in psychology, science and other (approximately 6**–**8% each). A descriptive analysis showed that, for all topics apart from PMH, we observed an increase over time in women authorship. Although PMH remained the dominant topic, there was a decline among women authors, with corresponding authorship dropping from 50.6% in 2016 to 39.6% in 2020, and last authorship decreasing from 52.6 to 45.2% (see Supplementary Fig. 1 and Supplementary 6 for further details).

There were changes over time as in 2016: men were predominant as corresponding authors in most fields and the largest discrepancy was in neurology and neuroscience (62.5%) but, by 2020, the gender gap had reversed in several fields, with women representing the majority of corresponding authors in MHP (64%), PMH (54%), psychology (76%), science (58%) and other (77%), with neurology and neuroscience becoming relatively balanced. A similar pattern was observed for first authors, with men being predominant in neurology and neuroscience (62%) and other (60%) but, by 2020, women accounted for the majority of first authors across all fields, including neurology and neuroscience (58%), and had particularly high representation in MHP (74%), PMH (60%) and other (83%). In regard to last authors, men were predominant in 2016 and continued to be so in 2020, although women did show a modest increase, particularly in psychology (58%) and science (55%), reflecting a trend towards greater women senior authorship in these disciplines. Some categories contained small numbers, restricting inferential gender comparisons.

A similar descriptive analysis of students (see Supplementary Table 7) showed that the number of publications had increased over time, with women producing more papers than men, especially as first author.

## Discussion

Our analyses, following the Athena Swan initiatives, showed that the increased number of women professors suggests that they had some effect, but barriers to career progression may still have existed in our observation period. In 2016, women are represented as corresponding and first authors but are less obvious in last-author positions. This was unaffected by the school where they were employed, but differences were observed across research topics, with greater gender balance in psychology but men predominating in both neuroscience and last-authorship positions. Over time, women were less likely to become last authors compared with men, even those with similar first-author publication records, suggesting potential gender biases or barriers in achieving senior authorship roles. This pattern suggests that structural barriers may hinder women’s progression to senior positions, potentially reflecting challenges in career advancement, mentorship access or institutional culture. First-author publications often serve as a stepping-stone to last authorship, which signifies a leadership role in research. This aligns with previous findings by Wykes et al,^4^ indicating persistent gender disparities despite the institutional effort that increased the number of women in professorial positions. Women were more highly represented as corresponding or first authors if they were students, suggesting that we do have an input of talented women who publish. However, given the results of the staff analyses, they do not progress to staff employment or, if they do, they become less represented as authors. The barrier at this point seems to be very high.

But there is good news. Our analyses of changes over time suggest a potential moderating effect of gender on the movement from first to last author from 2016 to 2018, but that this diminished between 2018 and 2020. This suggests that the institution’s actions have had some effect on career progression, even though this effect may have been small. For the research topic analyses, women’s representation increased over time, particularly as first and corresponding authors, with considerable gains in most topics but not in neuroscience; although representation increased in first and corresponding roles, it declined slightly for last authors. This decline in women’s last authorship suggests that some may leave academia before reaching senior roles, and future research is needed to assess whether mid-career attrition is a factor.

### Strengths and limitations

A key strength of this study is that it includes all available publications for the selected years, ensuring that the reported data fully represent IoPPN’s large research output. This guarantees that our findings are not based on a random sample and thereby avoid selection bias, sampling error and missing data, and ensure the reliability of observed trends in authorship. However, the study has some limitations. First, there is a risk of bias because individuals may have transitioned from first to last authorship during the intermediate years between 2016, 2018 and 2020. We have assumed that, because any such bias is similar across genders, schools and topics, the overall conclusions should remain valid. Second, we could not assess whether individuals who did not reach senior authorship had left academia, making conclusions about attrition speculative. Third, our binary gender classification may not have captured the full spectrum of gender identities. Fourth, papers published in 2020 might have been affected by COVID-19 but, given the lag time for publication, any impact is likely to be limited. Finally, without a comparison group or national-level data, we cannot determine whether observed trends are attributable to IoPPN’s Athena Swan policies or to broader, sector-wide changes, although the former is the most parsimonious explanation.

We have considered only gender in this paper, but there are other factors, particularly the intersection of different demographic attributes, that are likely to influence career progression – for instance, the intersection of ethnicity and gender that has been discussed elsewhere.^9,10^ Future investigations should consider these interacting factors that have been recognised in the newer Athena Swan policies.

Gender disparity in authorship roles still exists within mental health science, as it does across the health sciences generally,^4^ but there have been small improvements that should be celebrated. A comparison of this review and IoPPN figures over a similar period demonstrates that IoPPN had more women represented as first or last authors (first author 40.8 *v*. 63.9%, last author 36.7 *v*. 43.7%). The two results – observed changes in moderation effects and improvement in the topics – both indicate a potential reduction in the influence of gender over time. These small changes suggest that more nuanced factors might be influencing continuing disparities, with each factor playing a small but additive role in the barriers to career development.

In conclusion, this comprehensive investigation of gender inequality in academic publishing, within a single institution that subscribes to progressive policies, uncovered interesting results. While women are overrepresented as first authors, they remain underrepresented as last authors – a difference that persists over time, despite an increase in women’s employment even at senior levels. The institution’s interventions have often focused on structural barriers including parental support, promotion and wage inequality; these are likely to have effects on drop-out rates rather than directly affecting academic publishing. Although the effects of gender on transition to senior author have been small, they should be celebrated. Possible next steps include increasing the accountability of senior authors in promoting women into last-author roles and implementing targeted monitoring of authorship transitions alongside other career progression markers.

## Supporting information

Stahl et al. supplementary material Stahl et al. supplementary material

## Data Availability

The data that support the findings of this study are publicly available.

## Supplementary material

The supplementary material is available online at https://doi.org/10.1192/bjo.2025.10931
