# Supplementary material for: From first to last author: evaluation of women’s career progression in mental health publishing in one institution
Source: BJPsych Open. 2025 Dec 15;12(1):e17. doi: 10.1192/bjo.2025.10931 (PMC12724096; doi:10.1192/bjo.2025.10931)
Supplement: Stahl et al. supplementary material [file S2056472425109319sup001.docx]

**Supplement: IoPPN report - Are there changes in women’s authorship over time?**

Table of Contents

[**Data description by Topic and IoPPN Role** 2](#_Toc196403966)

[***Table S1:*** Topic hierarchy 2](#_Toc196403967)

[***Table S2*:** IoPPN Role hierarchy 5](#_Toc196403968)

[**Percentage and count data by Gender, Author Role, Time and School** 6](#_Toc196403969)

[***Table S3:*** percentage of IoPPN women staff by author role and time 6](#_Toc196403970)

[***Table S4:*** percentage of IoPPN women staff and students by author role and time 6](#_Toc196403971)

[***Table S5:*** percentage of IoPPN staff by gender, author role, time and School 7](#_Toc196403972)

[**Trends in publication count by IoPPN Role, Author Role and Time** 8](#_Toc196403973)

[***Figure F1*** 8](#_Toc196403974)

[**Trends in publication count by gender grouped by Author Role, Time and IoPPN School** 8](#_Toc196403975)

[***Figure F2*** 8](#_Toc196403976)

[***Figure F3*** 9](#_Toc196403977)

[***Figure F4*** 9](#_Toc196403978)

[**Trends in publication count by gender grouped by Topic and Time** 10](#_Toc196403979)

[***Figure F5*** 10](#_Toc196403980)

[***Table S6:*** gender distribution of roles by topics across years 12](#_Toc196403981)

[**Descriptive analyses of “student” publications** 13](#_Toc196403982)

[***Table S7****:* percentage of student publications by gender, author role, topic and time 13](#_Toc196403983)

# **Data description by Topic and IoPPN Role**

## ***Table S1:*** Topic hierarchy

| **Topic** | **ASJC codes** |
| --- | --- |
| *Psychiatry and Mental Health* | Biological Psychiatry  Psychiatric Mental Health  Psychiatry and Mental Health |
| *Psychology* | Applied Psychology  Clinical Psychology  Developmental and Educational Psychology  Experimental and Cognitive Psychology  General Psychology  Neuropsychology and Physiological Psychology  Psychology (miscellaneous)  Social Psychology |
| *Neurology and Neuroscience* | Behavioral Neuroscience  Cellular and Molecular Neuroscience  Cognitive Neuroscience  Developmental Neuroscience  General Neuroscience  Neurology  Neurology (clinical)  Neuroscience (miscellaneous) |
| *Medicine and Pharmacology* | Anatomy  Anesthesiology and Pain Medicine  Cancer Research  Cardiology and Cardiovascular Medicine  Dermatology  Emergency Medicine  Endocrine and Autonomic Systems  Endocrinology  Endocrinology, Diabetes and Metabolism  Gastroenterology  General Medicine  General Pharmacology, Toxicology and Pharmaceutics  Genetics (clinical)  Geriatrics and Gerontology  Gerontology  Health, Toxicology and Mutagenesis  Hematology  Hepatology  Immunology  Immunology and Allergy  Internal Medicine  Medicine (miscellaneous)  Microbiology (medical)  Molecular Medicine  Nephrology  Nutrition and Dietetics  Obstetrics and Gynecology  Oncology  Ophthalmology  Orthopedics and Sports Medicine  Otorhinolaryngology  Parasitology  Pathology and Forensic Medicine  Pediatrics, Perinatology and Child Health  Pediatrics, Peronatology and Child Health  Pharmaceutical Science  Pharmacology  Pharmacology (medical)  Physiology (medical)  Pulmonary and Respiratory Medicine  Radiology, Nuclear Medicine and Imaging  Reproductive Medicine  Rheumatology  Surgery  Toxicology  Transplantation  Urology  Virology |
| *Science* | Acoustics and Ultrasonics  Advanced and Specialized Nursing  Aging  Analytical Chemistry  Biochemistry  Biochemistry, Genetics and Molecular Biology (miscellaneous)  Biomedical Engineering  Biophysics  Biotechnology  Catalysis  Cell Biology  Chiropractics  Clinical Biochemistry  Development  Developmental Biology  Drug Discovery  Ecology  Electrical and Electronic Engineering  Environmental Chemistry  Epidemiology  General Agricultural and Biological Sciences  General Biochemistry,Genetics and Molecular Biology  General Chemical Engineering  General Chemistry  General Engineering  General Immunology and Microbiology  General Nursing  General Physics and Astronomy  Genetics  Immunology and Microbiology (miscellaneous)  Industrial and Manufacturing Engineering  Infectious Diseases  Inorganic Chemistry  Materials Chemistry  Maternity and Midwifery  Molecular Biology  Nursing (miscellaneous)  Oncology (nursing)  Organic Chemistry  Physical and Theoretical Chemistry  Physiology  Radiological and Ultrasound Technology  Sensory Systems  Spectroscopy  Speech and Hearing  Structural Biology |
| *Other* | Anthropology  Applied Mathematics  Artificial Intelligence  Arts and Humanities (miscellaneous)  Communication  Complementary and Manual Therapy  Computational Theory and Mathematics  Computer Graphics and Computer-Aided Design  Computer Networks and Communications  Computer Science (miscellaneous)  Computer Science Applications  Cultural Studies  Decision Sciences (miscellaneous)  Demography  Ecology, Evolution, Behavior and Systematics  Economics, Econometrics and Finance (miscellaneous)  Education  Family Practice  Food Science  Fundamentals and Skills  Gender Studies  General Decision Sciences  General Health Professions  General Social Sciences  Geography, Planning and Development  Health (social science)  Health Informatics  Health Information Management  Health Policy  Health policy, care planning  Health Professions (miscellaneous)  Health Science (social science)  Histology  History and Philosophy of Science  Human Factors and Ergonomics  Human-Computer Interaction  Information Systems  Information Systems and Management  Issues, Ethics and Legal Aspects  Language and Linguistics  Law  Leadership and Management  Library and Information Sciences  Life-span and Life-course Studies  Linguistics and Language  Management, Monitoring, Policy and Law  Modeling and Simulation  Multidisciplinary  Occupational Therapy  Organizational Behavior and Human Resource Management  Philosophy  Physical Therapy, Sports Therapy and Rehabilitation  Political Science and International Relations  Pollution  Public Administration  Public Health, Environmental and Occupational Health  Rehabilitation  Review and Exam Preparation  Safety, Risk, Reliability and Quality  Social Sciences (miscellaneous)  Sociology and Political Science  Software  Statistics and Probability  Statistics, Probability and Uncertainty  Strategy and Management  Theoretical Computer Science  Visual Arts and Performing Arts |

***Table S1:*** *shows how the ASJC codes were mapped into a predefined hierarchy of 5 topics.*

## ***Table S2*:** IoPPN Role hierarchy

| Type | Role |
| --- | --- |
| *Staff* | Academic  Clinical  Professional Services  Research |
| *Student* | Doctorate in Clinical Psychology (DClinPsych)  Doctor of Medicine (Research)  Doctor of Philosophy (PhD)  Master of Philosophy (MPhil)  student |

***Table S2:*** *presents how IoPPN roles were mapped into a predefined hierarchy or staff, student or visiting.*

# **Percentage and count data by Gender, Author Role, Time and School**

## ***Table S3:*** percentage of IoPPN women staff by author role and time

| **Role** | **Year** | **Women staff**  **N** | **% female authors** | **Total number of publications** |
| --- | --- | --- | --- | --- |
| **Corresponding** | 2016 | 154 | 56.8% | 261 |
|  | 2018 | 170 | 55.9% | 284 |
|  | 2020 | 189 | 58.2% | 323 |
| **First author** | 2016 | 185 | 63.4% | 256 |
|  | 2018 | 219 | 66.2% | 289 |
|  | 2020 | 237 | 64.8% | 315 |
| **Last author** | 2016 | 98 | 44.1% | 271 |
|  | 2018 | 92 | 41.8% | 242 |
|  | 2020 | 105 | 43.9% | 293 |

***Table S3*** *presents the number (N) and percentage (%) of women members of IoPPN who corresponding authors were, first authors or last authors in 2016, 2018 and 2020. Percentages refer to the proportion of women authors within each author roles and year. The table also shows the total number of publication for each role and year. It is important to note that the number of publications exceeds the number of unique authors due to some authors contributing to multiple publications.*

## ***Table S4:*** percentage of IoPPN women staff and students by author role and time

| Role | Year | Women Staff: authors | Staff % | Staff Unique papers | Women Student: Authors | Student % | Student: Number |
| --- | --- | --- | --- | --- | --- | --- | --- |
| Corresponding | 2016 | 132 | 55.2% | 237 | 22 | 68.8% | 24 |
|  | 2018 | 146 | 53.7% | 255 | 24 | 75.0% | 31 |
|  | 2020 | 166 | 57.2% | 296 | 23 | 65.7% | 28 |
| First author | 2016 | 138 | 60.3% | 198 | 47 | 74.6% | 60 |
|  | 2018 | 171 | 63.6% | 235 | 48 | 77.4% | 60 |
|  | 2020 | 188 | 63.9% | 263 | 49 | 68.1% | 62 |
| Last author | 2016 | 95 | 44.0% | 268 | 3 | 50.0% | 3 |
|  | 2018 | 92 | 42.0% | 242 | 0 | 0.0% | 0 |
|  | 2020 | 104 | 43.7% | 292 | 0 | 0.0% | 0 |

***Table S4*** *presents data from Table S1, separately for staff and student members,* *the number (N) and percentage (%) of women members of IoPPN who acted as corresponding, first or last authors in 2016, 2018 and 2020.*

## ***Table S5:*** percentage of IoPPN staff by gender, author role, time and School

| **Role** | **Year** | **Gender** | **School** | **N** | **%** |
| --- | --- | --- | --- | --- | --- |
| **Corresponding** | 2016 | f | MHaPS | 74 | 66.1% |
|  | 2016 | m | MHaPS | 38 | 33.9% |
|  | 2016 | f | Neuro | 22 | 38.6% |
|  | 2016 | m | Neuro | 35 | 61.4% |
|  | 2016 | f | Psych | 62 | 58.5% |
|  | 2016 | m | Psych | 44 | 41.5% |
|  | 2018 | f | MHaPS | 76 | 69.1% |
|  | 2018 | m | MHaPS | 34 | 30.9% |
|  | 2018 | f | Neuro | 29 | 39.2% |
|  | 2018 | m | Neuro | 45 | 60.8% |
|  | 2018 | f | Psych | 70 | 55.1% |
|  | 2018 | m | Psych | 57 | 44.9% |
|  | 2020 | f | MHaPS | 81 | 66.4% |
|  | 2020 | m | MHaPS | 41 | 33.6% |
|  | 2020 | f | Neuro | 27 | 40.9% |
|  | 2020 | m | Neuro | 39 | 59.1% |
|  | 2020 | f | Psych | 85 | 58.6% |
|  | 2020 | m | Psych | 60 | 41.4% |
| **First author** | 2016 | f | MHaPS | 82 | 68.3% |
|  | 2016 | m | MHaPS | 38 | 31.7% |
|  | 2016 | f | Neuro | 33 | 52.4% |
|  | 2016 | m | Neuro | 30 | 47.6% |
|  | 2016 | f | Psych | 74 | 65.5% |
|  | 2016 | m | Psych | 39 | 34.5% |
|  | 2018 | f | MHaPS | 91 | 75.2% |
|  | 2018 | m | MHaPS | 30 | 24.8% |
|  | 2018 | f | Neuro | 49 | 62.8% |
|  | 2018 | m | Neuro | 29 | 37.2% |
|  | 2018 | f | Psych | 85 | 61.2% |
|  | 2018 | m | Psych | 54 | 38.8% |
|  | 2020 | f | MHaPS | 86 | 62.3% |
|  | 2020 | m | MHaPS | 52 | 37.7% |
|  | 2020 | f | Neuro | 48 | 60.0% |
|  | 2020 | m | Neuro | 32 | 40.0% |
|  | 2020 | f | Psych | 108 | 68.8% |
|  | 2020 | m | Psych | 49 | 31.2% |
| **Last author** | 2016 | f | MHaPS | 54 | 60.7% |
|  | 2016 | m | MHaPS | 35 | 39.3% |
|  | 2016 | f | Neuro | 15 | 31.3% |
|  | 2016 | m | Neuro | 33 | 68.8% |
|  | 2016 | f | Psych | 31 | 35.6% |
|  | 2016 | m | Psych | 56 | 64.4% |
|  | 2018 | f | MHaPS | 43 | 55.1% |
|  | 2018 | m | MHaPS | 35 | 44.9% |
|  | 2018 | f | Neuro | 19 | 32.8% |
|  | 2018 | m | Neuro | 39 | 67.2% |
|  | 2018 | f | Psych | 32 | 36.8% |
|  | 2018 | m | Psych | 55 | 63.2% |
|  | 2020 | f | MHaPS | 47 | 59.5% |
|  | 2020 | m | MHaPS | 32 | 40.5% |
|  | 2020 | f | Neuro | 20 | 31.3% |
|  | 2020 | m | Neuro | 44 | 68.8% |
|  | 2020 | f | Psych | 38 | 39.6% |
|  | 2020 | m | Psych | 58 | 60.4% |

***Table S5: The number (N) and percentage (%) of male and female members of the IoPPN who acted as corresponding authors, first authors or last authors in 2016, 2018, and 2020, shown separately by school.***

# **Trends in publication count by IoPPN Role, Author Role and Time**

***Figure F1***

***Figure F1*** *Bar chart of the average number of publications by male and female staff and student members of the Institute of Psychiatry, Psychology & Neuroscience (IoPPN) who acted as corresponding authors, first authors, or last authors in the years 2016, 2018 and 2020.*

# **Trends in publication count by gender grouped by Author Role, Time and IoPPN School**

## ***Figure F2***

***Figure F2.*** *Bar chart of the relative number of women IoPPN members who were corresponding, first or last authors in 2016, 2018, and 2020, shown separately by School.*

***Figure F3***

***Figure F3****: Bar chart of the average number of publications by men and women staff members who served as corresponding authors, first authors, or last authors in the years 2016, 2018, and 2020 grouped by School.*

## ***Figure F4***

***Figure F4*** *is a similar plot to Figure S3, showing boxplots of the number of publications by men and women staff members who served as corresponding authors, first authors, or last authors in the years 2016, 2018, and 2020 grouped by School.*

# **Trends in publication count by gender grouped by Topic and Time**

## ***Figure F5***

***Figure F5:*** *bar charts which show the relative distribution of publication topics by corresponding, first, and last authors among men and women staff members of the Institute of Psychiatry, Psychology & Neuroscience for 2016, 2018 and 2020.*

***Table S6 shows the g****ender distribution of authorship roles across disciplines in 2016, 2018 and 2020. The table reports the number (N) and percentage of men and women listed as corresponding, first or last authors, stratified by six subject areas within the school. Percentages refer to the gender distribution within each author role, year and discipline. Note that authors may be counted more than once if they contributed to multiple publications.*

| Year | Gender | Author role | M,H & P | Neuro | P & MH | Psych | Science | Other | Total |
| --- | --- | --- | --- | --- | --- | --- | --- | --- | --- |
|  |  |  | N | N | N | N | N | N | N |
| 2016 | Women | Corresponding | 35 (47.3%) | 27 (37.5%) | 124 (53.4%) | 21 (58.3%) | 21 (48.8%) | 17 (58.6%) | 245 (50.4%) |
| 2016 | Men |  | 39 (52.7%) | 45 (62.5%) | 108 (46.6%) | 15 (41.7%) | 22 (51.2%) | 12 (41.4%) | 241 (49.6%) |
| 2018 | Women |  | 32 (42.7%) | 38 (42.7%) | 127 (53.1%) | 32 (74.4%) | 20 (51.3%) | 21 (70%) | 270 (52.4%) |
| 2018 | Men |  | 43 (57.3%) | 51 (57.3%) | 112 (46.9%) | 11 (25.6%) | 19 (48.7%) | 9 (30%) | 245 (47.6%) |
| 2020 | Women |  | 64 (64%) | 51 (47.7%) | 127 (54%) | 22 (75.9%) | 24 (58.5%) | 33 (76.7%) | 321 (57.8%) |
| 2020 | Men |  | 36 (36%) | 56 (52.3%) | 108 (46%) | 7 (24.1%) | 17 (41.5%) | 10 (23.3%) | 234 (42.2%) |
|  |  |  |  |  |  |  |  |  |  |
| 2016 | Women | First | 37 (58.7%) | 21 (38.2%) | 102 (57.6%) | 17 (73.9%) | 19 (63.3%) | 8 (40%) | 204 (55.4%) |
| 2016 | Men |  | 26 (41.3%) | 34 (61.8%) | 75 (42.4%) | 6 (26.1%) | 11 (36.7%) | 12 (60%) | 164 (44.6%) |
| 2018 | Women |  | 29 (54.7%) | 49 (68.1%) | 115 (57.2%) | 28 (84.8%) | 19 (55.9%) | 21 (65.6%) | 261 (61.4%) |
| 2018 | Men |  | 24 (45.3%) | 23 (31.9%) | 86 (42.8%) | 5 (15.2%) | 15 (44.1%) | 11 (34.4%) | 164 (38.6%) |
| 2020 | Women |  | 64 (74.4%) | 48 (58.5%) | 122 (60.4%) | 16 (64%) | 19 (63.3%) | 29 (82.9%) | 298 (64.8%) |
| 2020 | Men |  | 22 (25.6%) | 34 (41.5%) | 80 (39.6%) | 9 (36%) | 11 (36.7%) | 6 (17.1%) | 162 (35.2%) |
|  |  |  |  |  |  |  |  |  |  |
| 2016 | Women | Last | 32 (35.2%) | 28 (32.6%) | 142 (40.6%) | 28 (48.3%) | 19 (35.2%) | 21 (60%) | 270 (40.1%) |
| 2016 | Men |  | 59 (64.8%) | 58 (67.4%) | 208 (59.4%) | 30 (51.7%) | 35 (64.8%) | 14 (40%) | 404 (59.9%) |
| 2018 | Women |  | 36 (37.1%) | 25 (27.2%) | 123 (39.3%) | 24 (38.7%) | 18 (42.9%) | 19 (51.4%) | 245 (38.1%) |
| 2018 | Men |  | 61 (62.9%) | 67 (72.8%) | 190 (60.7%) | 38 (61.3%) | 24 (57.1%) | 18 (48.6%) | 398 (61.9%) |
| 2020 | Women |  | 46 (37.4%) | 39 (29.8%) | 135 (43.8%) | 28 (58.3%) | 23 (54.8%) | 28 (45.9%) | 299 (41.9%) |
| 2020 | Men |  | 77 (62.6%) | 92 (70.2%) | 173 (56.2%) | 20 (41.7%) | 19 (45.2%) | 33 (54.1%) | 414 (58.1%) |

# **Descriptive analyses of “student” publications**

## ***Table S7****:* percentage of student publications by gender, author role, topic and time

| Gender | Author | M,H & P | Neuro | P & MH | Psych | Science | Other | Total |
| --- | --- | --- | --- | --- | --- | --- | --- | --- |
| Men | Corresponding | 5 (9.8%) | 8 (15.7%) | 27 (52.9%) | 7 (13.7%) | 2 (3.9%) | 2 (3.9%) | 51 |
| Men | First author | 6 (6.3%) | 16 (16.8%) | 49 (51.6%) | 15 (15.8%) | 5 (5.3%) | 4 (4.2%) | 95 |
| Men | Last author | 3 (75%) | 1 (25%) | 0 (0%) | 0 (0%) | 0 (0%) | 0 (0%) | 4 |
| Women | Corresponding | 21 (21.9%) | 11 (11.5%) | 47 (49%) | 14 (14.6%) | 0 (0%) | 3 (3.1%) | 96 |
| Women | First author | 39 (18.8%) | 39 (18.8%) | 94 (45.4%) | 22 (10.6%) | 9 (4.4%) | 4 (1.9%) | 207 |
| Women | Last author | 1 (33.3%) | 2 (66.7%) | 0 (0%) | 0 (0%) | 0 (0%) | 0 (0%) | 3 |
|  | Total | 75 (16.5%) | 77 (16.9%) | 217 (47.6%) | 58 (12.7%) | 16 (3.5%) | 13 (2.9%) | 456 |

***Table S7:*** *shows the number of “student” publication topics (%) by corresponding, first, and last authors among women and men staff members of the Institute of Psychiatry, Psychology & Neuroscience (IoPPN) across 2016,2018 and 2020.*

**Table S7 shows the distribution of “student” publications across topic fields by gender and authorship position, combining data from 2016, 2018 and 2020. Women “**students” had a higher total number of publications (306) compared to men “students” (150), with the largest proportion of both groups publishing in Psychiatry & Mental Health (47.6% overall). Among corresponding authors, women “students” were listed 96 times as corresponding authors and published most frequently in Psychiatry & Mental Health (49.0%), followed by Mental Health & Psychology (21.9%). In contrast, men “students” had 51 publications as corresponding authors, with the highest proportion in Psychiatry & Mental Health (52.9%), followed by Neuroscience (15.7%).

First-author publications were more common among women “students” (207 vs. 95 for men). The proportion of Psychiatry & Mental Health publications was highest for both women (45.4%) and men (51.6%) first authors. Women “students” had a slightly higher proportion of first-author papers in Neuroscience (18.8%) compared to men (16.8%). Additionally, women “students” had a higher proportion of first-author publications in Mental Health & Psychology (18.8% versus 6.3% for men), whereas men “students” had a greater proportion of first-author papers in Psychology (15.8% for men vs. 10.6% for women).
